# Technologically Reflective Individuals as Enablers of Social Innovation*

**DOI:** 10.1111/jpim.12269

**Published:** 2015-05-20

**Authors:** Fiona Schweitzer, Christiane Rau, Oliver Gassmann, Ellis van den Hende

## Abstract

This paper identifies technologically reflective individuals and demonstrates their ability to develop innovations that benefit society. Technological reflectiveness (TR) is the tendency to think about the societal impact of an innovation, and those who display this capability in public are individuals who participate in online idea competitions focused on technical solutions for social problems (such as General Electric's eco‐challenge, the James Dyson Award, and the BOSCH Technology Horizon Award). However, technologically reflective individuals also reflect in private settings (e.g., when reading news updates), thus requiring a scale to identify them. This paper describes the systematic development of an easy‐to‐administer multi‐item scale to measure an individual's level of TR. Applying the TR scale in an empirical study on a health monitoring system confirmed that individuals' degree of TR relates positively to their ability to generate (1) more new product features and uses, (2) features with higher levels of societal impact, and (3) features that are more elaborated. This scale allows firms seeking to implement co‐creation in their new product development (NPD) process and sustainable solutions to identify such individuals. Thus, this paper indicates that companies wishing to introduce new technological products with a positive societal impact may profit from involving technologically reflective individuals in the NPD process.

**Practitioner Points**

For practitioners, this paper

- develops an easy‐to‐administer instrument to systematically identify an individual's level of technological reflectiveness.
- demonstrates the benefits of integrating technologically reflective individuals in the concept generation and refinement phase for technology‐triggered social innovation.
- outlines ways in which technologically reflective individuals can contribute to diverse innovation tasks.

## Introduction

Technical solutions pervade everyday life (Majchrzak and Markus, 2013) and have improved life in many respects; for example, information and communication technology (ICT) has made shopping easier (e.g., Amazon), citizens more powerful (e.g., the Arab spring), and information more accessible (e.g., Google) (Geoghegan, Lever, and McGimpsey, 2004). Technical solutions are changing social interaction patterns (e.g., Facebook, Twitter) and work routines (e.g., mobile customer relationship management) (Thurner and Chaffey, 2013). For more than three decades, socio‐technical research has stressed the interrelatedness and interconnectedness of social and technical systems (e.g., Bostrom and Heinen, 1977; Cohen, 2010; van de Poel, 2003). Technology masterminds such as Bill Gates believe that technology has the power to transform society and solve many of today's social problems (Gates, 2013). Nevertheless, not all technical solutions serve the higher good. For example, chips in printers that can supposedly distinguish original equipment manufacturers' cartridges from clone cartridges and manipulate the printing performance accordingly (Anderson, 2008) are not likely to serve higher social goals.

Social innovations are innovations geared toward solving social issues, such as improving health care, protecting the environment, and reducing poverty (Barczak, 2012). Relatively few academic studies have examined technical products' potential to contribute to solving these social challenges (Seidel, Recker, and vom Brocke, 2013). Very little is known about the best practices, methods, processes, or particular skills and abilities that might be necessary to develop technical innovations that serve to solve social issues (Barczak, 2012) and can be understood as “technology‐enabled social innovations.”

This paper focuses on technologically reflective individuals' ability to contribute to technology‐enabled social innovation and defines technological reflectiveness (TR) as an individual's tendency to think about the impact of a technological product on its users and society in general. Technologically reflective individuals reflect privately (e.g., when reading the news at home), in a review (e.g., peer to peer, manager to team member, master–apprentice relationship), or more publicly (e.g., commenting on technology articles, answering questions on dedicated innovation pages in online communities).

Such thoughts are beneficial for new product development (NPD), since technologically reflective individuals often develop their thoughts into suggestions to improve technology‐enabled social innovations. For example, there is a growing set of new social entrepreneurs, includes companies like Causehub, Makani Power, and Samasource, which develop technical solutions to social problems (Small, 2014). Arthur Zang, a computer engineer and a typical representative of people who turn their reflective practice into social entrepreneurship, is a 2014 Rolex Award Laureate (i.e., an award for inspiring individuals who carry out innovative projects that advance human knowledge or well‐being). Zang developed the touch screen Cardio Pad, which allows reliable diagnosis and cardiac care in remote regions where access to electricity to run sophisticated medical equipment is scarce. He developed Cardio Pad from an intrinsic motivation to use computers' potential to solve social challenges and change the lives of others for the better (Rolex, 2014). Similarly, Benjamin Cohen, cofounder and CEO of Tohl's, a nonprofit organization that delivers clean water with solar‐powered pipelines to people in emergency situations, shows dedication to helping suffering people. He persevered until he found a technical solution that would provide a social benefit (Nee, 2014).

Moreover, cocreation challenges are examples that demonstrate the relationship between TR and developing solutions for social innovations. Participation rates in online idea competitions, such as General Electric's eco‐challenge and the BOSCH Technology Horizon Award, attest to a growing number of individuals with an affinity for socio‐technical reflection, who, in a next step, are willing to share their social innovation ideas. So far, however, innovation literature has ignored the potential of these individuals. This paper argues that technologically reflective individuals' tendency to think about technology and society enables them to identify the opportunities and challenges that technical products are likely to present and pose for society.

The TR concept extends the stream of innovation research that investigates the influence of individual trait‐based qualities on users' contribution to new product development (see Table 1 for an overview). The lead user concept has dominated this body of literature (von Hippel, 1986). In recent years, researchers have investigated further traits that either extend the lead user construct (e.g., Schweisfurth and Raasch, 2015), or are discrete traits (e.g., Wellner and Herstatt, 2014). For example, Hoffman, Kopalle, and Novak (2010, p. 855) developed the emergent nature scale and demonstrated its value by showing that individuals ranking high on this scale have a “unique capability to imagine or envision how concepts might be further developed so that they will be successful in the mainstream marketplace.” Nevertheless, trait‐based approaches are still underexplored, and the selection of the right type of user to involve in different tasks throughout the innovation process remains challenging (Biemans and Langerak, 2015).

**Table 1:** Overview of Selected Trait‐Based NPD Studies

Categories of Relevant User Traits for Involving Users in NPD	Representative Studies
**Lead userness**	von Hippel, 1986; Lüthje and Herstatt, 2004; Schreier and Prügl, 2008; Lilien et al., 2002
**Extended lead‐user traits**	
Embedded lead users Technical lead users	Schweisfurth and Herstatt, 2015; Schweisfurth and Raasch, 2015 Lettl, Rost, and von Wartburg, 2006; Lettl, 2007
**Other user traits**	
Emergent user Entrepreneurial users Users of local information Users with product expertise Users with technical expertise	Hoffman, Kopalle, and Novak, 2010 Dahlin, Taylor, and Fichman, 2004; Hienerth, 2006 Lüthje, Herstatt, and von Hippel, 2005 Schoormans, Ortt, and de Bont, 1995; Schweitzer, Gassmann, and Rau, 2014 Wellner and Herstatt, 2014

This paper develops and validates a scale to measure TR. This TR scale provides an easy to administer instrument to systematically identify an individual's level of TR. Following the approach by Hoffman et al. (2010), the scale validation steps demonstrate that individuals with high levels of TR have the ability to produce ideas that improve the societal impact of technical products.

The remainder of this paper unfolds as follows: first, it elaborates on the concept of technologically reflective individuals by distinguishing it from related terms. Second, drawing from the literature on domain‐specific skills, it explains why technologically reflective individuals are in a good position to improve technology‐enabled social innovations. Third, the development of the TR scale follows (Study 1 to Study 4a). Fourth, the paper tests the TR scale's discriminant and nomological validity, and demonstrates that this scale successfully measures technology reflectiveness and discerns between individuals with differing ability to provide improvement suggestions for technology‐enabled social innovations (Study 4b to Study 7). Fifth, a general discussion of the findings follows, including their contribution to innovation management research and practice, and suggestions for topics for future research.

### Theoretical Localization of TR

TR is an individual's tendency to think about a technological product's impact on its users and society in general. TR draws from theories on reflection (e.g., Boud, Keogh, and Walker, 1985; Trapnell and Campbell, 1999) and reflexivity (e.g., Hammedi, van Riel, and Sasovova, 2011).

#### TR as an act of reflection

Reflective thinking is a disposition to engage in repetitive thinking about an issue based on epistemic curiosity, i.e., a philosophical love of thinking about actions. In psychology, self‐reflection is a particular type of often investigated reflection (e.g., Grant, Franklin, and Langford, 2002; Harrington and Loffredo, 2011). Though geared toward the self, it offers insights into reflective behavior's outcomes that are relevant for TR geared toward society at large. Self‐reflection is thus the process of constructively examining oneself and one's actions in a certain situation; it is motivated by interest in the self and by the desire to understand the reason for certain behavior, as well as the emotions and consequences (Trapnell and Campbell, 1999). Self‐reflective individuals analyze their behavior retrospectively, try to understand this behavior, and consider alternative actions. This reflective behavior allows them to gain deep insights into problems and to develop advanced problem‐solving strategies (Harrington and Loffredo, 2011; Kross, Ayduk, and Mischel, 2005; Lyubomirsky, Tucker, Caldwell, and Berg, 1999).

The term TR relates to self‐reflection in as far as both have a tendency to indulge in a retrospective reflection process. TR differs from self‐reflection by not being directed at the self and one's actions, but at the technology–society link. Technologically reflective individuals analyze the past effects of technological products on society, contemplate technological solutions' potential effects on society, and might develop an advanced understanding of socio‐technical relationships.

#### TR as the concurrence of action and thinking

Schön (1983) enriched the reflection construct through his studies on reflective practitioners by introducing the reflection in action concept. Reflection in action describes the concurrence of action and thinking. Further, it can be interpreted as a specific form of mindfulness (Jordan, Messner, and Becker, 2009), or need for cognition (Cacioppo and Petty, 1982). In turn, mindfulness is “the state of being attentive to and aware of what is taking place in the present” (Brown and Ryan, 2003, p. 822). To reframe a situation and redirect behavior, reflection‐in‐action practitioners use a repertoire of past experiences and understanding to actively examine their assumptions and patterns of interaction in an unfolding situation (Mezirow, 1994). Similarly, technologically reflective individuals use their past experience with technological products and their past reflections on socio‐technical issues to judge and evaluate technological concepts that are new to them, which in turn lead to a self‐reinforcing learning process. Contrary to reflection in action, which focuses on managerial practices, TR focuses on understanding a technological product's impact on its users and society.

#### TR as an act of reflexivity on methods and processes

Reflexivity is “the opposite of blindly accepting and applying a given method” (Hammedi et al., 2011, p. 663). Within the reflexivity concept, reflecting on a certain subject or action raises awareness of its appropriateness, suitability, and limitations within a certain context (West, 1996). Reflexivity is often studied in the form of team reflectiveness (e.g., Hammedi et al., 2011; Schippers, Den Hartog, and Koopman, 2007), and is believed to lead teams to adjust their methods, criteria, processes, or actions in order to improve their decision‐making (Schippers et al., 2007). Differing from reflection, reflexivity is not limited to quiet thought or contemplation, but is understood as a group process in which the appropriateness of certain tools, processes, and actions in terms of attaining aspired goals is overtly discussed within the team (West, 1996). For example, innovation teams, who evaluate, openly discuss, and communicate the suitability of screening methods and criteria, have been found to improve screening processes' efficiency and effectiveness (Hammedi et al., 2011).

Similar to reflexivity, TR can be regarded as a cognitive effort directed at a subject of interest (the impact of technology on society) and question specific artifacts', or situations', appropriateness and quality for the subject of interest. Unlike team reflexivity, TR is not restricted to open group discussions, but can also take place in an introspective format.

#### TR and other technology‐centered traits

TR differs from technology‐oriented constructs, such as technical skills (e.g., Benaroch, Lichtenstein, and Robinson, 2006), technical optimism (Parasuraman, 2000), and technical innovativeness (Agarwal and Prasad, 1998). The term *technical skills* comprises knowledge of technical issues, as well as the ability to deploy and use this knowledge to solve technical problems (Benaroch et al., 2006; Hulland, Wade, and Antia, 2007). Although technically skilled people might reflect more than average individuals on technology, because they are more often confronted with technology, they do not necessarily also reflect more on questions regarding technology's impact on society. Technical skills are clearly different capabilities than TR, as the former concentrates on technical understanding and the latter on the understanding of socio‐technical interrelations.

Technical optimism is the tendency to have positive feelings about a technology (Parasuraman, 2000). Technical innovativeness is a term used in a consumer context to distinguish early adopters of technical innovations from more cautious buyers of new technical products (Agarwal and Prasad, 1998; Goldsmith and Hofacker, 1991). While technical innovativeness and technical optimism have a clearly positive inclination toward technology, TR's direction is neutral. Technologically reflective individuals can see the positive and the negative aspects of technology; they are not necessarily “geeks” nor “technophobes,” but can be euphoric, or skeptic, about a certain technical innovation, or about technology in general. What matters is that they think about technology's impact on society.

### The Role of TR Individuals in Driving the Societal Benefit of Technical Products

Technologically reflective individuals have a natural inclination to consider technical and social systems, as well as their interactions. They reflect more intensively and more often on technology and society than others. By doing so, they strengthen their reflecting skills regarding technology and society, thus developing expertise in this domain. These domain‐specific skills supposedly enable technologically reflective individuals to clearly see technological products' potential positive and negative societal impacts and to develop suggestions for improving their societal contributions.

Domain‐specific knowledge, or expertise, is the amount and quality of knowledge that an individual has of a specific domain (Amabile, 1996; Ericsson, Charness, Feltovich, and Hoffman, 2006). Individuals with high domain‐specific knowledge perform better than those with low domain‐specific skills regarding cognitive tasks (e.g., Chi, Feltovich, and Glaser, 1981; Ericsson and Klintsch, 1995) and intuitive tasks (e.g., Pham, Lee, and Stephen, 2012) within this domain. To solve these tasks, individuals with domain‐specific knowledge draw consciously, or unconsciously, from prior experience in and knowledge of the domain (Ericsson and Klintsch, 1995; Pham et al., 2012).

Ericsson and Klintsch (1995) and Chi et al. (1981) find that people with high domain‐specific knowledge are better and faster at absorbing, storing, and recalling new information in this domain. Pham et al. (2012) find that familiarity with a certain knowledge domain enables individuals to better generate solutions and solve intuitive tasks in this domain by merely relying on their feelings than those without such expert knowledge. This positive trust‐in‐feelings effect allows experts to unconsciously and holistically access the broad body of knowledge they have of this domain (Pham et al., 2012). Closer to the topic of this paper, Schweitzer, Gassmann, and Rau (2014) indicate that the type of newly generated product ideas depends on the area in which individuals possess domain‐specific skills.

### Scale Development (Studies 1–4a) and Scale Validation (Studies 4b–7)

The following first presents the TR scale development studies (Study 1 to Study 4a) and, subsequently, the TR scale validation studies (Study 4b to Study 7). All the studies were conducted in German.^1^

The objective of Study 1 to Study 4a was to develop a reliable scale to identify individuals' TR levels. Scale development used four steps, following the recommendations of Churchill (1979), and Netemeyer, Bearden, and Sharma (2003): Study 1 generated an empirically based understanding of the aspects that technically reflective individuals show. Study 2 generated items and selected the most viable in terms of their content validity. Study 3 checked whether the wording was suitable for the general audience and for a particular group of technologically reflective individuals. Finally, Study 4a refined the scale and assessed its reliability by testing it with 1000 respondents.

### Study 1: Explorative Analysis

In Study 1, 10 experts in the fields of psychology, sociology, and innovation management received the conceptual definition of technologically reflective individuals (i.e., individuals who think about technical innovations' impact on society). In semi‐structured interviews, they had to describe the characteristics and behaviors of technologically reflective individuals. In these interviews, the main questions were: Which attitudes and characteristics do technologically reflective individuals exhibit in your opinion? How would you recognize a technologically reflective individual? Which behavior (e.g., information behavior), activities, or lifestyle would you consider typical of technologically reflective individuals? Which skills do such individuals have? What could the reflecting process comprise? Do technologically reflective individuals in your opinion differ from ethical consumers, technology optimists/pessimists, or individuals who are socially critical? Why? How far? Qualitative content analysis revealed an extensive set of 118 preliminary aspects related to the TR construct.

### Study 2: Item Generation, Selection, and Content Validity

At the next stage, the content validity of these aspects was assessed by consulting with five researchers, who actively conduct quantitative research in the area of innovation management. The experts were given the definition of the TR construct and asked to rate the aspects with regard to their representativeness of the construct's domain on a 3‐point scale (i.e. “not representative,” “somewhat representative,” and “clearly representative”). Aspects with a low level of agreement were dropped (at least three evaluators needed to agree to retain an aspect in the proposed scale). This process helped refine the understanding of the TR's theoretical construct, followed up by reduction of ambiguous, contradictory aspects, split double‐barred aspects, and integrated similar aspects.

The analysis revealed three main facets central to the TR concept. The first facet pertains to the motivation to think about the interaction between technology and society. Technologically reflective individuals are motivated to seriously consider different aspects of a technology and enjoy thinking through their potential implications. They are willing to take a differentiated look at technologies' impact. Further, they are more likely to appreciate the complexity of interdependencies and are disposed to take many factors into consideration. The second facet relates to technologically reflective individuals' ability to think beyond their personal product usage. They somewhat include potential personal uses in their product thinking, but simultaneously mainly consider the product's relevance for other persons and social groups.

The third facet relates to the capabilities needed to make sophisticated assessments about a technology's potential implications. The experts stress that technologically reflective individuals reflect more often on socio‐technical issues than other individuals do. Their constant practice develops and reinforces their capability. Moreover, due to their interest, these individuals actively collect information on potential technological effects, and observe which effects actually manifest over time. Their active cognition and observation of how technology changes society, together with their growing knowledge base, reinforce and sharpen their ability to assess future developments.

Beside these three main facets, four additional aspects that describe technologically reflective individuals were identified (i.e., “taking a clear position,” “general cognitive abilities,” “width of the scope of interest,” and “being able to understand another person's perspective”). While these aspects occur intensively in technologically reflective individuals, they are not included in the TR construct as they are not relevant. All four aspects are predispositions that support individuals to develop TR, but are not part of TR itself. The decision to discard these four aspects for the final item generation was made on the basis of discussions between the first and the second author concerning the scope of the scale, its comprehensiveness, and its preciseness. Following up on the aim to focus on measuring technology reflectiveness ability resulted in discarding general individual abilities, characteristics, and behaviors.

In line with standard practice, there was a tendency to err toward being over inclusive in the early exploratory stages (DeVellis, 2003). The final set of agreed‐upon facets of the technical reflectiveness construct built the bases for the item generation: a preliminary pool of 25 items after the exploratory stage. The aim was to achieve a balance between keeping the length of the scale parsimonious and also maintaining the underlying theoretical conceptualization. The reasonable length of this scale reduces potential study costs and respondent fatigue (Steenkamp and Baumgartner, 1995). All the items were constructed using simple, straightforward language that avoided trendy expressions, or colloquialisms, which established guidelines on item writing recommend (e.g., Angleitner and Wiggins, 1985; Kline, 1986). For content validation purposes, a panel of six scientists with a quantitative research background in psychology and the social sciences was asked to check the items for language, readability, and general presentation. This exercise resulted in minor language rephrasing.

### Study 3: Qualitative Pilot Study

Following inputs from the previous study, this study proceeded toward validating the 25 items of the proposed TR scale with potential respondents: a convenience sample of 10 individuals to check the items for language, readability, and general presentation. Eight of the respondents provided feedback either personally, or by phone, while two respondents provided written feedback. The respondents pointed toward potentially ambiguous formulations and provided suggestions for further simplification of items. The feedback served as input for slight modification and refinement of some items.

### Study 4a: Assessment of Scale Reliability

Study 4a presented the 25 items to 1000 randomly selected respondents from a well‐reputed market research institution's online panel. The items were randomly rotated to avoid potential order bias, and the respondents answered each item on a 7‐point scale (ranging from 7 = “strongly agree” to 1 = “strongly disagree”) to assess the individual's level of TR. While expecting a second‐order construct of TR with the three facets, (1) motivation to think about the interaction between technology and society, (2) ability to think beyond one's personal product usage, and (3) the capabilities needed to make sophisticated assessments about a technology's potential implications, an exploratory factor analysis revealed high loadings of the three conceptualized TR facets on the first component (eigenvalues 13.954; 1.252; 1.108), and the break in eigenvalues indicated a unidimensional structure. The three factors explained 65.254% of the common variance in the items. Second‐order factor analyses showed poor results (x^2^/df = 10.158; incremental fit index [IFI] = .870; Tucker‐Lewis index [TLI] = .857; confirmatory fit index [CFI] = .870). Therefore, the scale development continued by using the items of each facet instead of using the originally intended separate subscales for each facet. This step was necessary to provide a short scale that is easy to administer in surveys that use different personal trait scales to screen participants, or in studies where TR is not the core concept under study (Kline, 1986). The item reduction procedure as outlined by Spiro and Weitz (1990) ensured a unidimensional scale with items that fulfill the following criteria: (1) highly intercorrelated items (ITTC > .6), (2) items representing all three facets, (3) items loading highly on the first principal component (exploratory factor loadings [EFL] > .7). This procedure resulted in the final seven‐item scale presented in Table 2a, 2b. The final scale contains two items of facet 1 (item 1 and item 2), three items of facet 2 (item 4, item 6, and item 7), and two items of facet 3 (item 3 and item 5). The final TR scale explained 68.77% of the common variance in the items (eigenvalue 4.813), with exploratory factor loadings above .70, indicating convergent validity (Churchill, 1979). The Cronbach's alpha of .924 implied high internal consistency, while the item‐to‐total correlations (ITTCs) ranged between .646 and .814. The final scale also performed well in the confirmatory factor analyses (CFAs), with an average variance extracted (AVE) of .637, a composite reliability (CR) of .924, and all the CFA factor loadings positive and significant (Netemeyer et al., 2003). The means (M), standard deviations (SD), reliability measures, and confirmatory and exploratory factor loadings of all the variables of the final TR scale are presented in Table 2a, 2b.

**Table 2a:** Items and Characteristics of Technological Reflectiveness in Study 4a to Study 7

Item	Study 4a (*n* = 1,000) *α* = .924; AVE = .637, CR = .924				Study 4b (*n* = 502) *α* = .927 ; AVE = .649; CR = .928				Study 5 (*n* = 274) *α* = .935; AVE = .678; CR = .936			
	Mean	SD	ITTC	CFL	Mean	SD	ITTC	CFL	Mean	SD	ITTC	CFL
1. I enjoy thinking about the chances and risks a new technology might provide and harbor for society.	3.97	1.71	.814	.858	4.05	1.69	.833	.874	4.09	1.59	.847	.880
2. I am very interested in studying the impact new technical products have on society.	4.64	1.67	.646	.668	4.69	1.66	.666	.693	4.52	1.57	.708	.759
3. When I hear about a new technical product, I spontaneously have ideas on how this product can be used to reduce social problems.	3.80	1.58	.777	.822	3.86	1.53	.773	.815	3.73	1.60	.754	.784
4. I enjoy thinking about the impact that new technical products have on different social groups (e.g., the elderly, the young, and the chronically ill).	4.10	1.68	.800	.834	4.15	1.66	.797	.827	4.25	1.57	.823	.847
5. When I hear that a new technical product is on the market, I immediately reflect on the consequences this product may have for society.	3.87	1.66	.713	.746	3.96	1.59	.708	.739	4.01	1.66	.771	.803
6. I enjoy thinking about ways in which future technology could change our society.	4.37	1.71	.782	.802	4.42	1.67	.811	.835	4.30	1.61	.822	.850
7. I often think about how technical products could impact the autonomy and self‐determination of individuals and social groups.	3.98	1.72	.792	.842	4.07	1.68	.793	.839	4.10	1.60	.806	.833

*α*, Cronbach's alpha; AVE, average variance extracted; CFL, factor loadings in confirmatory factor analysis; CR, composite reliability; ITTC, item‐to‐total correlations; SD, standard deviation.

**Table 2b:** Items and Characteristics of Technological Reflectiveness in Study 4a to Study 7

Item	Study 6 (*n* = 253) *α* = .885; AVE = .528; CR = .886				Study 7 (*n* = 134) *α* = .893, AVE = .535, CR = .888			
	Mean	SD	ITTC	CFL	Mean	SD	ITTC	CFL
1. I enjoy thinking about the chances and risks a new technology might provide and harbor for society.	3.72	1.664	.739	.745	3.91	1.71	.709	.775
2. I am very interested in studying the impact new technical products have on society.	4.78	1.677	.626	.787	4.90	1.72	.647	.691
3. When I hear about a new technical product, I spontaneously have ideas on how this product can be used to reduce social problems.	3.47	1.555	.600	.678	3.77	1.54	.600	.566
4. I enjoy thinking about the impact that new technical products have on different social groups (e.g., the elderly, the young, and the chronically ill).	3.72	1.622	.701	.753	3.92	1.69	.724	.754
5. When I hear that a new technical product is on the market, I immediately reflect on the consequences this product may have for society.	3.75	1.570	.630	.655	3.84	1.65	.683	.720
6. I enjoy thinking about ways in which future technology could change our society.	4.06	1.765	.735	.672	4.09	1.78	.755	.811
7. I often think about how technical products could impact the autonomy and self‐determination of individuals and social groups.	3.80	1.732	.692	.782	3.99	1.81	.725	.775

### Study 4b: Preliminary Nomological Validity

Study 4b assesses the preliminary nomological validity of the TR scale (items scores in Table 2a, 2b), which is extended in Study 6 and Study 7. As a preliminary indicator of the scale's appropriateness and nomological validity, a random sample of 502 of the 1000 respondents of Study 4b (see above) received an additional task on the impact of ICT on the autonomy of individuals in a society after answering the initial 25 items of the TR scale. More specifically, the respondents got five minutes to list examples of how the Internet can be used to increase/reduce the autonomy of individuals. The analysis used a count of the number of negative and positive examples that each respondent listed, to test its correlation with the TR scale. A positive relationship was expected between TR and the number of enumerated advantages and disadvantages, as this is an intuitive indicator of an individual's ability to assess a technology's impact on society. A Pearson's correlation coefficient of .325 (p < .001) demonstrated considerable positive correlation. While acknowledging the possible confound created by the carry‐over effect, this result is consistent with the posited theoretical expectation and offers the first support for the scale's nomological validity. Study 7 adopts a methodology to separate the assessment of TR and the dependent variable and finds strong support for nomological validity.

### Study 5: Test–Retest Reliability and Discriminant Validity 1

Study 5 examined the test–retest reliability of the seven‐item TR scale by subjecting these items to a random sample of 300 individuals from Study 4a (who did not receive the additional task presented in Study 4b). The retest included 274 fully completed responses (91.3% response rate) and was carried out four months after Study 4a, using the same market research institution, to allow enough time for memory effects to abate. By comparing the individuals' TR scale scores in the test (Study 4a) and retest situation (Study 5), it is possible to test whether their answers were stable over time (individual TR item scores in Table 2a, 2b). The TR scale shows high test–retest reliability (*r* = .759, p < .001), supporting its stability.

In addition to the TR scale, Study 5 included scales to measure creativity (Hoffman et al., 2010), the need for cognition (Cacioppo and Petty, 1982), general optimism (taken from the revised life orientation test by Scheier, Carver, and Bridges, 1994), self‐reflection (Martin and Debus, 1999), and technology optimism (adapted from the technology optimism items of Parasuraman, 2000, so that the items would better reflect the context of the present study: 1. In general, I am optimistic about the performance of technical products. 2. I trust that technical products will increasingly improve mankind's quality of life. 3. Generally speaking, I think that new technical products make mankind more independent and self‐determined. 4. I think that new technical products will increase their users' physical and mental capabilities).

To determine the reliability and discriminant validity of the new TR scale, calculations of Cronbach's alpha, ITTCs, and the exploratory factor loadings of the TR scale and the five other scales in the study were followed up by a CFA by means of AMOS (IBM SPSS AMOS 20.0, AMOS Development Corporation, Meadville, PA, USA). Table 3 reports the results of the analyses, as well as the means and standard deviations. As shown in Table 3, the Cronbach's alpha values of the six constructs used in this study (TR, creativity, need for cognition, general optimism, self‐reflection, and technology optimism) were all above the .70 threshold, ranging between .823 and .947. The factor loadings of the CFA of the six constructs were between .648 and .953. To assess the discriminant validity of the TR scale, the TR measure must differ from creativity, the need for cognition, technology optimism, self‐reflection, and general optimism. The fit indices confirmed a six‐factor solution (x^2^/df = 541.017/260; IFI = .939; TLI = .929; CFI = .939; root mean square error of approximation [RMSEA] = .063), while the fit of a single‐factor structural model was poor (x^2^/df = 2549.236/275; IFI = .505; TLI = .409; CFI = .500; RMSEA = .172). Further, as shown in Table 3, the AVE was greater than the squared correlations between each of the constructs, demonstrating discriminant validity, and supporting TR as constituting a separate scale distinct from other constructs (Fornell and Larcker, 1981).

**Table 3:** Means, Standard Deviations, Correlations, and Square Root of AVE (*n* = 274) in Study 5

	Mean	SD	No Items	*α*	ITTC	CFL	CR	1	2	3	4	5	6
1 Creativity	4.558	1.509	3	.947	.862–.913	.897–.953	.947	(.926)					
2 General optimism	4.261	1.384	4	.833	.590–.740	.648–.857	.836	.396**	(.751)				
3 Need for cognition	2.815	1.305	3	.836	.664–.735	.738–.863	.837	−.248**	−.088	(.795)			
4 Technology optimism	4.554	1.272	4	.898	.720–.800	.780–.861	.899	.344**	.206**	−.141*	(.831)		
5 Self‐reflection	4.417	1.226	4	.823	.623–.675	.668–.782	.823	.367**	.250**	−.079	.248**	(.733)	
6 Technological reflectiveness	4.147	1.359	7	.935	.708–.847	.759–.880	.936	.569**	.538**	−.129*	.216**	.404**	(.823)

* *p* < 0.05; ** *p* < 0.01; Square root of average variance extracted (AVE) is shown on diagonal in parentheses.

*α*, Cronbach's alpha; CFI, comparative fit index; CFL, factor loadings in confirmatory factor analysis; CR, composite reliability; IFI, incremental fit index; ITTC, item‐to‐total correlations; RMSEA, root mean square error of approximation; SD, standard deviation; TLI, Tucker‐Lewis index.

Fit indices in confirmatory factor analysis: *x^2^*/df = 541.017/260; IFI = .939; TLI = .929; CFI = .939; RMSEA = .063.

### Study 6: Nomological Validity 1 and Discriminant Validity 2

#### Goal

The first goal of Study 6 was to test the antecedents of TR in order to provide evidence of nomological validity by following the procedure outlined by Tian, Bearden, and Hunter (2001). Perspective taking (i.e., the ability to adopt other people's point of view, Davis, 1980) and technical skills (i.e., the ability to solve technical problems, Benaroch et al., 2006) might be antecedents that explain why some individuals tend to be more technologically reflective than others. The second goal of Study 6 was to perform a further test of discriminant validity to ensure that the new TR construct differed from two consumer type constructs that, in prior studies, have proved central for selecting consumers with a high ability to contribute valuable information for innovation tasks (i.e., lead users and users with an emergent nature) (Hoffman et al., 2010; von Hippel, 1986).

#### Method

A quota‐sampling procedure resulted in 253 participants. The sample consisted of 52.6% women (47.4% men). The median age class was 40–44 (on an answer scheme ranging from 20 to 80 in constant age classes of five years per class), the median net monthly income ranged between EUR 1200 and EUR 1799, and 47% of the participants had completed secondary education (53% held a college degree or a higher post‐secondary education level).

#### Measures

The study used 7‐point scales and adapted established measures for the two antecedents: (1) perspective taking (by Davis, 1980; M = 4.734; SD = 1.144; *α* = .735), and (2) technical skills. For technical skills, the study adapted Franke, von Hippel, and Schreier's (2006) technical expertise scale with the general product category of household devices as the focal product category, because the scale required this (M = 3.996; SD = 1.696; *α* = .902; 1. I can repair my own household devices. 2. I can help other people solve problems with household devices. 3. I can make technical changes to my household devices on my own. 4. I am handy and enjoy tinkering. 5. I come from a technical background in my profession or education (e.g., engineering). 6. I am an enthusiast of the technical aspects of household devices).

In addition, the second discriminant validity test employed the emergent nature scale by Hoffman et al. (2010) (M = 3.984; SD = 1.315; *α* = .911) and adapted their lead user scale to capture lead users of health‐oriented household devices (M = 1.623; SD = .984; *α* = .823; 1. Other people consider me as “leading edge” with respect to health‐oriented household devices. 2. I have pioneered some new and different ways of checking and improving health through household devices. 3. I have suggested to companies or organizations some new and different ways of checking and improving health through household devices. 4. I have come up with some new and different solutions to meet my needs for health improvement and control at home).

#### Results and discussion

Similar to Study 5, the key data in 2a, 2b, 4 ensured the reliability and the discriminatory validity of all the constructs. Cronbach's alphas, ITTCs, and the exploratory and confirmatory factor loadings were all above the required thresholds, except for one item on the perspective‐taking scale, which had an exploratory factor loading of .58. Discriminant validity was tested by calculating the fit of the single‐factor model (x^2^/df = 3649.739/700; CFI = .518; RMSEA = .105), the five‐factor solution (x^2^/df = 1316.827/680; CFI = .90; RMSEA = .049), and by applying the Fornell–Larcker criterion. With the five‐factor model showing good fit, the single‐factor model showing bad fit, and the Fornell–Larcker test showing AVE greater than the squared correlations, the discriminant validity of all the constructs was ascertained.

**Table 4:** Means, Standard Deviations, Correlations, and Square Root of AVE (*n* = 253) for Study 6

	Mean	SD	No. of Items	*α*	ITTC	CFL	CR	1	2	3	4	5
1. Technological reflectiveness	3.900	1.274	7	.885	.600–.739	.655–.787	.886	(.726)				
2. Perspective taking	4.734	1.144	3	.735	.479–.609	.581–.750	.743	.266**	(.703)			
3. Technical skills	3.996	1.696	6	.902	.667–.807	.710–.861	.908	.304**	−0.122	(.789)		
4. Lead userness	1.623	0.984	4	.823	.607–.784	.643–.953	.755	.263**	−0.060	.278**	(.716)	
5. Users with an emergent nature	3.984	1.315	8	.911	.664–.776	.702–.815	.913	.628**	.194**	.428**	.232**	(.753)

*n* = 253; ** *p* < 0.01; *α*, Cronbach's alpha; CFI, comparative fit index; CR, composite reliability; RMSEA, root mean square error of approximation; SD, standard deviation.

Square root of average variance extracted (AVE) is shown on diagonal in parentheses. Fit indices in confirmatory factor analysis: *x^2^*/df = 1316.827/680; CFI = .90; RMSEA = .049.

After ensuring reliability and discriminant validity, nomological validity was checked, by regressing the perspective‐taking and the technical skills on TR. Results showed that both perspective‐taking (standardized beta coefficient [std. *β*] = .358, *p* < .001) and technical skills (std. *β* = .379, *t* = *p* < .001) contribute to explaining the extent of an individual's TR (x^2^/df = 430.970/202; CFI = .926, RMSEA = .054). These affirmative results demonstrate that the TR scale possesses distinct antecedent causes, to which TR is related to a differing quantitative extent, thus demonstrating nomological validity (Tian et al., 2001).

### Study 7: Nomological Validity 2

#### Goal

The goal of Study 7 was to further test the TR scale's nomological validity, thus completing the series of scale development studies. More precisely, this study proved the TR scale's ability to provide useful input for product concept tests (i.e., wanted to demonstrate its predictive validity, see the procedure outlined by Tian et al., 2001). The study measured TR two weeks before the actual study to minimize carry‐over effects (as addressed in Study 4b). Furthermore, the study tested if individuals with high values on the TR score can enumerate more suggestions for increasing a product concept's positive societal impact. Last, while Study 4b demonstrated TR's nomological validity in the context of the Internet and an individual's autonomy, the present study wanted to demonstrate it in a second context (i.e., a health monitoring device). Thus, a new product category was tested to demonstrate the TR scale's broad applicability.

#### Method

Study 7 included 134 participants, selected by means of a quota‐sampling procedure. The sample consisted of 54.5% women with a median age class of 40–44 (on an answer scheme ranging from 20 to 79 in constant age classes). Two weeks prior to the main study, the respondents received a questionnaire measuring TR, and demographics such as gender and age. Two weeks later, the same respondents visited the lab and were instructed to read a text about a product concept for a health monitor that records and displays eating habits, work‐out, and health data. The device can take blood samples and has various internal and external sensors for measurements. As the device also includes ICT, it exchanges data online with a medical service where a doctor that analyzes the data and provides suggestions (via written reports and online consulting sessions) for improving the user's health. This e‐health product concept was chosen because it potentially affects a number of societal groups and their understanding of the possible interactions. This ICT product has notable effects on health‐care systems (including doctors, patients, and insurance companies) and addresses several important societal challenges (e.g., obesity, medical care cost, and data privacy issues). Hence, it offers room for controversial debate, potentially stimulating the enumeration of the advantages and the disadvantages, and offers the possibility to suggest concept improvements.

The respondents were given 10 minutes to list concept improvement suggestions (“Please write down new features and/or new uses for the health monitor concept. Please focus on suggestions for different health care and medical functions/services that improve the health care system [e.g., use the health monitor to exchange information on blood sugar values between diabetics and their doctors]”). This task resulted in the following dependent variables (see Measures section): (1) the number of enumerated improvement suggestions (NEIS), and (2) the elaboration of enumerated improvement suggestions (EEIS). The two variables represented the quantitative (NEIS) and qualitative (EEIS) elements of the ability to produce ideas for improving a new product concept's societal impact (McGill and Anand, 1989; Valgeirsdottir, Onarheim, and Gabrielsen, 2014).

Further, to build a nomological network (Tian et al., 2001), the respondents completed the questionnaire with variables, which, in past research, had been found to be essential for solving creative technical challenges (i.e., general creativity and product involvement [Amabile, 1996]).

#### Measures

In Study 7, the average score on the TR scale (see Table 2a, 2b for the seven items) was 4.029 (SD = 1.317). The TR scale (Cronbach's alpha = .893) explained 61.1% of the variation in the correlations of the single items. A confirmatory factor analysis showed an AVE of 0.535 and a CR of 0.888 (for an overview of the scales used in Study 7, see Table 5; for details of the TR items see Table 2a, 2b). Further, comparisons were made between the square root of the average variation extracted (AVE) and the correlation between each of the other variables. As shown in Table 5, the square root of the AVE was greater than the correlations, demonstrating discriminant validity (Fornell and Larcker, 1981).

**Table 5:** Means, Standard Deviations, Correlations, and Square Root of AVE (*n* = 134) in Study 7

	Mean	SD	1	2	3	4	5	6
1 Gender (Dummy)	.545	.500						
2 Age	5.560	3.323	−.013					
3 Product involvement	4.878	1.273	.058	.195*	(.754)			
4 General creativity	4.739	2.604	.139	−.094	.014			
5 TR	4.029	1.317	−.073	−.144	.210*	.269**	(.731)	
6 NEIS	3.925	3.023	.196*	−.389**	.024	.404**	.387**	
7 EEIS	43.351	34.638	.105	−.456**	−.030	.333**	.431**	.718**

* *p* < 0.05; ** *p* < 0.01; SD, standard deviation; Square root of average variance extracted (AVE) is shown on diagonal in parentheses (where applicable), technical reflectiveness (TR) and product innovativeness are factors, TR with *α* .893; CR .888; AVE .535 (see also Table 2a, 2b) and product innovativeness with *α* .917; CR .913; AVE .568; number of enumerated improvement suggestions (NEIS), elaboration of enumerated improvement suggestions (EEIS).

The two dependent measures were constructed as follows: To determine the NEIS, the number of improvement suggestions that each respondent mentioned was summed up (M = 3.925; SD = 3.023). The length of the statements served as a measure for EEIS through a count of the words in the statements (M = 43.351; SD = 34.638) (McGill and Anand, 1989).

The alternative use task measure of the Torrance Test of Creative Thinking measured general creativity. The respondents got two minutes to list as many different uses for a common brick as they could think of (Torrance, 1990). To use this measure in the analyses, the number of alternative uses that each respondent generated in this task was counted (M = 4.739; SD = 2.604), high numbers representing more creative individuals. The product involvement measure adopted Zaichkowsky's (1994) personal involvement inventory (7‐point scales; M = 4.878; SD = 1.273; *α* = .917; AVE = .568; CR = .913).

#### Results and discussion

After calculating the means and standard deviations as reported in Table 5, all the values were standardized and the further analyses used z‐scores to facilitate the interpretation (Mahr, Lievens, and Blazevic, 2014). Testing for the TR scale's predictive validity, two hierarchical regression models, one for each of the dependent variables (NEIS, EEIS), were used. Both hierarchical regressions used age, gender, product involvement, and general creativity as independent variables. Second, TR was added to the first analysis (see Nenkov, Inman, and Hulland, 2007 for a similar approach). The results presented in Table 6 demonstrate that TR significantly predicted both dependent variables. The first hierarchical regression model demonstrated that TR had a significant and positive effect (std *β* = .295, *t* = 3.718) on the number of improvement suggestions that individuals were able to generate (NEIS). When compared to the regression without TR (step 1, left column), the increase in *R^2^* (Δ*R^2^* = .072) and the significant changes in *F* (Δ*F* = 13.820, *p* [Δ*F*] < .001) regarding the regression analyses with TR (step 2, right column) further supported the effect of TR on the NEIS. Similarly, the results showed a significant and positive effect for TR in Model 2 regarding the EEIS (*β* = .357, *t* = 4.455, Δ*R^2^* = .106, Δ*F* = 19.850, *p* [Δ*F*] < .000). Models 1 and 2 demonstrate that individuals with higher TR produce more (NEIS) and better (EEIS) suggestions for improving the investigated ICT and the health monitoring product concept's societal impact. These results indicate that the ability to generate ideas for ICT‐triggered social innovations increases with individuals' degree of TR, which cannot be accounted for by existing constructs, such as general creativity and product involvement.

**Table 6:** Regression Results in Study 7

	Model 1 number of enumerated improvement suggestions (NEIS)		Model 2 elaboration of enumerated improvement suggestions (EEIS)	
	Beta (T)	Beta (T)	Beta (T)	Beta (T)
Gender	.171 (2.220)*	.202 (2.724)**	.093 (1.169)	−.131 (1.748)
Age	−.283 (−3.572)***	−.225 (−2.905)**	−.321 (−3.920)***	−.250 (−3.202)**
Product involvement	.027 (.355)	−.047 (−.618)	.009 (.114)	−.077 (−1.011)
General creativity	.314 (3.970)***	.246 (3.164)**	.246 (3.013)**	.162 (2.065)*
TR		.295 (3.718)***		.357 (4.455)***
*R^2^*	.257	.330	.210	.316
Changes in *R^2^*		.072		.106
Changes in *F*	11.169	13.820	8.582	19.850
Significant changes in *F*	.000	.000	.000	.000

* *p* < 0.05; ** *p* < 0.01; *** *p* < 0.001.

## General Discussion

This paper identified individuals who have a tendency to think about the impact of a technological product on its users and society in general. It argued and demonstrated that these individuals can improve new product concepts, such as health‐care solutions. This paper contributes to the innovation management literature with a new and validated trait‐based scale that focuses on the important technology and society relationship. With this scale, firms can identify individuals who are better able to produce valuable improvement suggestions. Previous research investigated the influence of individual trait‐based qualities on an individual's contribution to different activities in the NPD process (e.g., Ettlie and O'Keefe, 1982; Kirton, 1976; Mansfeld, Hölzle, and Gemünden, 2010), but none of these examined individuals who are able to reflect on the impact of technology or technological products on society. The findings indicate that TR enriches the literature that aims to understand which individuals are effective contributors to technical innovations by taking society into account.

The study results provide innovation managers striving to develop technology‐enabled social innovations with the means to do so, but also those who want to increase the social contribution of technical products being developed. In the light of companies' increasing endeavors to open up the innovation process and seize the abilities and skills of external sources in the innovation process (Gassmann, 2006), the new easy‐to‐administer TR scale can be used to recruit externals with high‐technology reflectiveness scores to contribute to the innovation process. Given these findings, specifically at the fuzzy front‐end, externals with high TR scores might be tapped to improve product concepts. If they are integrated into the innovation process early on, they can shape the product concepts in a socially beneficial way, potentially diminishing barriers to market adoption. If taken to heart, their suggestions might compensate—at least partially—for corporate blind spots in the area of social responsibility by ensuring that technical concepts' social consequences are holistically reviewed.

This paper's findings are subject to limitations that provide avenues for further research. The performance of technologically reflective individuals was examined in two contexts only: the Internet in Study 4b and e‐health in Study 7. Other contexts, such as sustainability and ICT, could be included in future research.

The paper examined the contribution of technologically reflective individuals to concept refinement in the early stages of the innovation process, which could be interpreted as limited NPD tasks and limited outcomes. In Study 4b, the respondents had to enumerate how the Internet can be used to increase or reduce individuals' autonomy. In Study 7, the respondents had to provide suggestions for improvement of the concept's positive societal impact. Further research should examine whether technologically reflective individuals perform equally well in respect of different NPD tasks. For example, it might be interesting to study the impact of technologically reflective individuals on beta version launches (e.g., of e‐health apps). Furthermore, the outcome performance measure could be broadened; research might profitably examine technologically reflective intellectual capital's effect on the competitive advantages of firms.

The studies used very strict time limits for the task that the respondents had to perform, which had proved successful in similar studies (e.g., Kristensson and Magnusson, 2010). Individuals rating above the median on the TR scale produced an average of 2.23 ideas and 24.43 words more than individuals rating below the median. Although this difference is significant (*t* = 4.673, p < .01 for number of ideas; *t* = 4.334, *p* < .01 for number of words), the strict time limit may have restricted technologically reflective individuals' ability to live up to their full capacity, as this is not similar to their usual reflection practice, when they take all the time they need to reflect on such issues. Future studies could investigate the performance of technologically reflective individuals with tasks that are not time restricted.

The validation studies were carried out in online settings (Study 4b, Study 5, and Study 6) and in a quasi‐experimental laboratory setting (Study 7) with consumers. Nevertheless, TR could also be an important employee trait. For, example, in the context of sustainable NPD, Esslinger (2011, p. 401) points toward “designers' responsibility to connect and coordinate human needs and dreams with new opportunities and inspirations from science, technology, and business in order for products and their usage to be culturally relevant, economically productive, politically beneficial, and ecologically sustainable.” While this is certainly true for designers, it might also be important for organizational innovation teams and innovation managers. In this respect, future studies could use the TR scale to analyze the effect of employees' technological reflectiveness on a company's ability to develop technology‐triggered social innovation.

Overall, the results in this paper suggest that identifying and using individuals with high TR is an important contribution to current innovation research and is worth exploring in further research in order to more fully understand their contribution to the entire NPD process.

## Footnote

1 The TR scale was developed in German and applied in experimental settings with German‐speaking participants. Two bilingual professional language teachers (native English and native German), following the procedure suggested by Shoham and Ruvio (2008), translated the items to English and back to German to ensure a valid translation of the TR scale items for publication. In consultation with one of the authors, the native English‐speaking professor evaluated the translation for clarity, content‐applicability equivalence, and general understandability. Minor disagreements were resolved by reaching consensus with the authors. One author translated all the other scales used in the studies, with a bilingual language professor rechecking this translation.
